# Inferring What to Do (And What Not to)

**DOI:** 10.3390/e22050536

**Published:** 2020-05-11

**Authors:** Thomas Parr

**Affiliations:** Wellcome Centre for Human Neuroimaging, University College London, 12 Queen Square, London WC1N 3BG, UK; thomas.parr.12@ucl.ac.uk

**Keywords:** Bayesian, information gain, experimental design, inference, message passing, anatomy, active inference

## Abstract

In recent years, the “planning as inference” paradigm has become central to the study of behaviour. The advance offered by this is the formalisation of motivation as a prior belief about “how I am going to act”. This paper provides an overview of the factors that contribute to this prior. These are rooted in optimal experimental design, information theory, and statistical decision making. We unpack how these factors imply a functional architecture for motivated behaviour. This raises an important question: how can we put this architecture to work in the service of understanding observed neurobiological structure? To answer this question, we draw from established techniques in experimental studies of behaviour. Typically, these examine the influence of perturbations of the nervous system—which include pathological insults or optogenetic manipulations—to see their influence on behaviour. Here, we argue that the message passing that emerges from inferring what to do can be similarly perturbed. If a given perturbation elicits the same behaviours as a focal brain lesion, this provides a functional interpretation of empirical findings and an anatomical grounding for theoretical results. We highlight examples of this approach that influence different sorts of goal-directed behaviour, active learning, and decision making. Finally, we summarise their implications for the neuroanatomy of inferring what to do (and what not to).

## 1. Introduction

An important recent development in the study of motivated behaviour is the idea of planning as inference [1,2]. In place of asking how we *decide* what we will do next, we ask how we *infer* what we will do. This simple reformulation recruits the toolbox of statistical inference in service of understanding motivation. Motivational drives are then simply the things that contribute to our prior beliefs about how we will act [3]. These priors are updated to posterior beliefs based upon sensory observations that afford evidence for or against alternative hypothetical behaviours. Characterising motivational drives now becomes a problem of specifying a prior distribution over the ways in which we might expect an individual to behave. Here, we review attempts to find the constituents of this prior. Broadly, these are divided into exploratory and exploitative influences [4]. Exploratory drives are grounded in information theory and experimental design [5,6,7,8], while exploitative influences are frequently encountered in control problems in engineering [9,10]. We hope to provide some intuition as to these contributing factors. However, the purpose of this article is not to provide principled derivations for each of these contributions. Instead, our aim is to consider their influence over behaviour, the consequences of their disruption, and their biological substrates.

Understanding the form of a prior belief tells us about the dependencies between variables in a model or, more accurately, between the probability distributions describing these variables. This dependency structure may be interpreted as a neuronal network [11,12], expressing how populations of neurons interact with one another. While offering a plausible computational architecture for planning as inference, much of our work is still ahead of us. The outstanding challenge is to associate this (behaviourally motivated) connectivity structure with the known anatomy of the nervous system. This is where perturbations of these systems, either experimentally or following pathological insult, offer solutions. If we understand the behavioural consequences of perturbations to a computational neuronal network, we can use observations of those same behavioural consequences in biological systems to constrain our understanding of functional anatomy.

Given their importance in planning, we highlight the role of frontal cortical and basal ganglia regions [13,14,15]. We consider the role of connections between the frontal and posterior cortices in contextualising information-seeking behaviour, the influence of the medial prefrontal cortices in determining preferences, the role of the direct and indirect pathways of the basal ganglia in promoting and suppressing policy execution, and the role of the nigrostriatal system in modulating the balance between these pathways. This paper is organised as follows. Section 2 provides an overview of inference and message passing in graphical models. Section 3 unpacks information-seeking behaviour in terms of optimal experimental design and information gain. Section 4 focuses on utility-based formalisms. Section 5 incorporates information seeking and utility into a prior belief about actions, based upon an expected free energy measure. Section 6 and Section 7 then deal with extensions of this in deep models and in the optimisation of precision (or confidence). Throughout, we consider the neurobiological manifestations of these ideas and the experimental perturbations that help to constrain the computational neuroanatomy of planning as inference.

## 2. Graphical Models and Inference

We start with a brief review of Bayesian inference and its relevance to graphical (generative) models [16,17,18,19,20,21]. The idea here is that sensory observations (*o*) may be explained by states of the world (*s*) that generate them. As such, if we have a forward model that tells us the likelihood of making an observation conditioned upon each possible state of the world and prior beliefs about these states, we can use Bayes’ theorem to compute the posterior probability of states given the observation we make:

(1) P(o|s)P(s)=P(o,s)=P(o)P(s|o)

The left-hand side of Equation (1) shows the product of a likelihood and prior. This is equivalent to the joint probability of observations and states (shown after the first equality) and the product of the marginal likelihood of observations (also known as Bayesian model evidence) with the posterior probability of states given observations (shown on the right-hand side). The marginal likelihood may be seen as an objective function for Bayesian statistics and, more broadly, for autonomous self-organising or “self-evidencing” [22] systems. Self-evidencing systems are those who act to maximise Bayesian model evidence for their internal model of the world. As evidence is a function of data, this means acting to change sensory input to be consistent with that anticipated under a model. This is a very general way of describing systems that maximise an objective function defined in relation to sensory input, as such objectives may be reinterpreted as un-normalised log probability measures. In addition, it offers a normative standard for theoretical research that we implicitly appeal to throughout this article [23]. It will be convenient in what follows to use a softmax functional (or its continuous equivalent) to express these relationships more concisely:

(2) $$\begin{array}{c} \;\;\;\;\;\;\;\;\;\;P(s|o)=\sigma_{s}\left[\ln P(o,s)\right]\\ \;\;\;\;\;\;\;\;\;\;P(o|s)=\sigma_{o}\left[\ln P(o,s)\right]\\ \sigma_{a}\left[b(a)\right]≜\frac{\exp\left(b(a)\right)}{Z}\\ \;\;\;\;\;\;\;\;\;\;\;Z≜\left\{ \begin{array}{l} \int \exp\left(b(a)\right)da,\;\;if\;a\in \mathbb{R}\\ \sum_{a}\exp\left(b(a)\right),\;\;if\;a\in \{0,\ldots ,N\} \end{array}\right. \end{array}$$

The softmax functional (*σ*) exponentiates and normalises its argument. We will use a subscript to indicate which variable we are normalising with respect to. Normalisation ensures consistency with a probability distribution, which must sum to one. In what follows, we will sometimes deal with data that have already been observed, and sometimes with data that have yet to be observed. We will use *o*_<_ to mean data that have already been collected and will occasionally use the shorthand *Q*(*s*) = *P*(*s*| *o*_<_) for concision.

**Figure 1:**
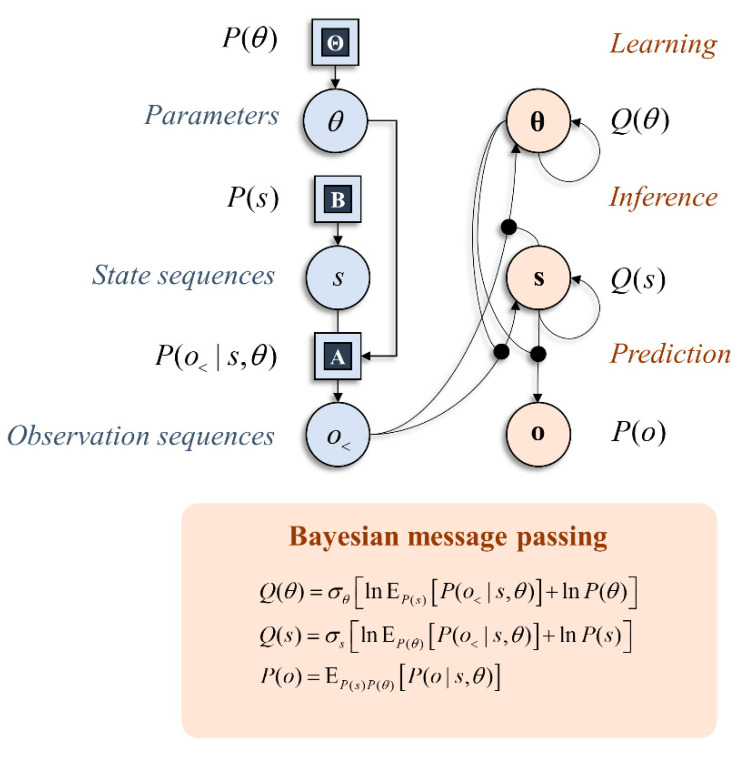
This schematic illustrates inference through message passing. The graphic on the left in blue shows a factor graph expressing a simple generative model. This shows factors of a generative model as squares and variables in circles. The generative model shown here says simply that observations (*o*) are generated by hidden states (*s*) via a likelihood distribution with parameters (*θ*). The graphic on the right shows that this model may be inverted to find the probability of states given past observations (*o*_<_), as indicated by the arrow from the *o*_<_ circle to the **s** circle, and prior beliefs about states, as indicated by the arrow from **s** to **s**. The former depends upon beliefs about the parameters of the likelihood mapping—this is shown through the circular arrowhead going from **θ** to the connection between the *o*_<_ circle and the **s** circle. Similarly, beliefs about parameters are updated based upon prior beliefs about parameters and observations contextualised by beliefs about states. We will consistently use bold in subsequent figures to represent sufficient statistics of probability distributions.

With these preliminaries in place, we can start to think about the implications this has for the neural architectures that solve particular types of inference problem. Figure 1 shows a simple graphical representation of a generative model and an interpretation of the computations required to find posterior distributions in terms of the passing of local messages [11,12,24]. The graphical representation on the right shows the dependencies implicit in the Bayes optimal updating of beliefs. This is divided into three parts: learning, inference, and prediction [25]. Neurobiologically, we can interpret the relationship between learning and inference as the distinction between neuronal firing and synaptic plasticity. The change in post-synaptic neuronal firing (**s**) in response to pre-synaptic sensory input (*o*_<_) depends upon the synaptic gain (**θ**). Similarly, changes in synaptic efficacy (under Hebbian assumptions [26,27]) depend upon the pre- and post-synaptic activity. The importance of prediction will become clearer in the next section, where we consider the role anticipated sensory data have on alternative behavioural strategies. Practically, the computations shown in Figure 1 rely upon (mean-field) approximations that simplify the message passing. However, our primary focus here is on characterising priors for policy selection, and we will gloss over the details of these inference schemes and assume exact Bayesian inference is tractable. While there may be subtle differences resulting from the application of different message-passing schemes [11,24,28], this will not influence the computational anatomy at the level of description adopted in this paper, which rests upon conditional dependencies or Markov blankets in a generative model. Markov blankets are statistical constructs that partition sets of random variables. A Markov blanket of a subset of variables comprises the variables outside this subset that, if known, would render all other variables uninformative about the subset of interest [29].

## 3. Information and Experimental Design

### 3.1. Mutual Information

An important aspect of behaviour is the drive towards exploration or information seeking [30]. This is sometimes framed as “active learning” [31] or “active inference” [25]. Simply put, curiosity influences what we do. This tells us that we have a prior belief that those policies leading to greater information gain are more probable. As such, we need to find a way of scoring the amount of information available under each policy. Fortunately, information theoretic measures have been developed for this purpose [6,32,33]. Specifically, they arise in the field of optimal experimental design [5]. If we are trying to design the best scientific experiment, our aim is to collect those data that best inform our beliefs about alternative hypothetical states of the world. Formalising this, the optimality of an experimental design can be quantified through the mutual information (ℐ) between the hypotheses and observations recorded as a consequence of the experiment (*π*):

(3) $$ℐ(\pi )=D_{KL}\left[P(o,s|\pi )||P(o|\pi )P(s|\pi )\right]$$

The mutual information scores the KL-Divergence or relative entropy between the joint distribution of states and observations under a given experiment or policy, and the product of the marginal distributions of states and observations. This is the average of the log ratio of two probability distributions. It is greater than zero unless the two distributions are equal. If states and observations are independent of one another under a given policy, these distributions will be identical and the mutual information will be zero. This implies a poor experiment, as the observations made under that design are irrelevant as far as our beliefs about states are concerned. This has been most intensively studied in the setting of active vision [33,34,35,36], where saccadic eye movements are seen as experiments to gather visual data [33,37]. Empirically, the importance of Equation (3) in human behaviour has been demonstrated through eye-tracking studies [38,39], which showed that visual foraging is better explained with this quantity than without it. Mutual information can be further interpreted in various ways through two alternative factorisations of the first argument. The first of these yields an expression in terms of the expected divergence between a prior and posterior distribution:

(4) $$\begin{array}{c} P(o,s|\pi )=P(s|o,\pi )P(o|\pi )\\ \;\;\;\;\;\;\;\;\Rightarrow ℐ(\pi )=E_{P(o|\pi )}D_{KL}\left[P(s|o,\pi )||P(s|\pi )\right] \end{array}$$

Equation (4) says that the expected information gain can be thought of as the divergence between our prior beliefs and the posterior beliefs we expect following a new observation. In other words, it is the degree to which we believe we will update our beliefs if we perform an experiment (or behave a certain way). The alternative factorisation lets us express the mutual information as the difference between two entropies:

(5) $$\begin{array}{c} P(o,s|\pi )=P(o|s,\pi )P(s|\pi )\\ \;\;\;\;\;\;\;\;\Rightarrow ℐ(\pi )=E_{P(o,s|\pi )}\left[\ln P(o|s,\pi )-\ln P(o|\pi )\right]\\ \;\;\;\;\;\;\;\;=H\left[P(o|\pi )\right]-E_{P(s|\pi )}\left[H\left[P(o|s,\pi )\right]\right] \end{array}$$

Shannon entropy (*H*) is defined as an average negative log probability. It is a measure of the dispersion or uncertainty in a probability distribution. Equation (5) is useful in expressing the important aspects of good experimental design. The first term expresses the uncertainty in the data anticipated following an experiment. The more uncertain we are, the better the experiment. Intuitively, there is no point in performing an experiment if we already know what the data will look like. The second term subtracts the conditional entropy of observations given states. If this uncertainty is very high, it means that there is a great deal of noise in the process generating observations from states. This would be a poor experiment as we could not be confident that our data would be informative about their causes. By subtracting the conditional entropy (or ambiguity) from the total predictive uncertainty, we are left with the resolvable uncertainty—i.e., information gain.

### 3.2. Attention Networks and Disconnection

To understand the biological substrates of information-seeking behaviour, it is worth thinking about situations in which this is impaired. A cardinal example of this is visual neglect—a neuropsychological syndrome in which the left-hand side of space is ignored relative to the right [40]. Neglect has been demonstrated in a variety of paradigms [41,42,43,44,45], including in the domain of saccadic eye-movements. This failure of left-sided exploration may be understood by considering the function of the brain’s attention networks [46]. These networks comprise the areas connected by a white matter tract known as the superior longitudinal fasciculus [47,48,49,50] that runs between the front (frontal cortex) and back (temporal and parietal cortices) of the brain. This tract is divided into three parts. The first of these connects dorsal frontal regions associated with gaze direction to dorsal regions of the posterior cortices. The second connects the same frontal areas to more ventral posterior cortices. The third connects ventral regions in the frontal and posterior cortices.

Interpreting this functionally, in terms of the graphic in Figure 1, if we associate dorsal frontal regions with beliefs (**s**) about gaze direction, it follows that these areas must be connected to regions representing the sensory consequences (*o*) of gaze-direction, with the synaptic efficacies of neurons mediating these connections encoding beliefs about parameters (**θ**). Clearly, the sensory consequences are largely in the visual domain. This is consistent with the frontal–posterior connectivity of the first two branches of the superior longitudinal fasciculus, as it is the posterior cortices that are most associated with early visual pathways. Activity in dorsal visual pathways is associated with where an object in visual space is located, while activity in ventral pathways is associated with the identity of that object. The implication here is that the first and second branches of the fasciculus correspond to the likelihood distribution that predicts where and what (respectively) is seen conditioned upon a gaze-direction. In other words, the second branch is the structural manifestation of “what I would see if I looked there” [51].

**Figure 2:**
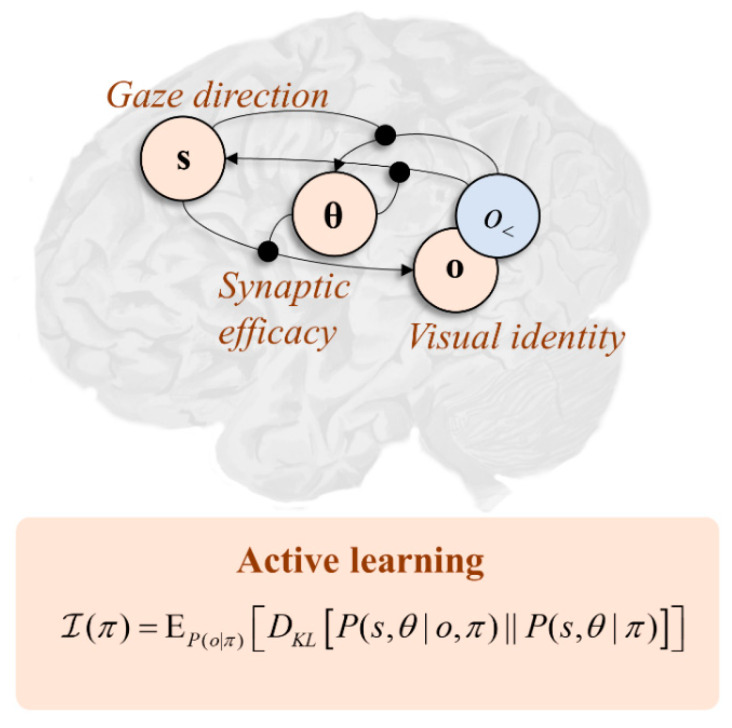
The graphic in this figure shows the anatomy that underwrites the saccadic exploration of a visual scene. This is framed in terms of the connections between the dorsal frontal cortex and more ventral regions of the posterior cortices (i.e., those neurons whose axons run in the second branch of the superior longitudinal fasciculus). When these connections are damaged in the right hemisphere, this causes visual neglect, a condition in which the left-side of space is ignored. The “active learning” panel offers an explanation for why this is. When an axon is cut, the efficacy associated with that connection can no longer be changed by making new observations. Scoring eye-movements (*π*) in terms of their information gain, this means there is little point looking at the left side of space as there is a greater expected change in efficacy associated with saccades to the right.

Under this view, the experiments we could perform comprise alternative eye-movements, which alter the gaze direction. Clearly there will not be a great deal of uncertainty to resolve about the gaze-direction itself, as this is precisely determined by (dorsal) visual and proprioceptive sensory data. However, there is still uncertainty in beliefs about the parameters encoding beliefs about what will be seen at each location. This is supported by evidence that the coupling between these areas is modified during the visual exploration of simple arrays [52]. Extending the expected information gain to beliefs about parameters (see Figure 2), this implies eye movements that maximally alter the efficacy of synapses between dorsal frontal and ventral posterior cortical regions (i.e., of those axons travelling in the second branch of the superior longitudinal fasciculus) afford the most promising experiments. Under the view that the brain selects behavioural policies that maximise information gain, this provides a clear explanation for neglect syndromes [53,54]. If we disconnect the fasciculus in the right hemisphere, it becomes impossible to change the efficacy of the (now absent) connections. This implies any saccades towards the left visual field—depending upon the right frontal cortex—are poor experiments relative to rightward saccades. This provides a possible explanation for an important clinical syndrome and constrains the computational anatomy of active learning.

## 4. Utility and Steady State

### 4.1. KL-Control

The formulation above deals with a purely curious agent, who suffers no consequences and derives no extrinsic value from the outcomes it encounters (e.g., [55]). However, this is clearly not applicable to real creatures, who benefit from those outcomes in a distribution conducive to survival and are at risk from those that deviate from this [56]. Drawing from statistical physics [57,58,59], biological systems exist at non-equilibrium steady state. This means that there is a distribution over the states in which we expect to find them that stays consistent over time (at least, at a given timescale). Any deviation from this steady state is corrected by a move back towards more probable configurations. This has the appearance of goal-directed behaviour, in the sense that creatures act to ensure consistency with this distribution. The association between observations that are probable under steady state and “goals” rests upon the (circular) definition of goals as simply those things we act to attain [60,61].

The implication of the above from a motivational point of view is that we can score alternative behaviours in terms of their consistency with the steady state distribution. This means we should penalise those policies for which the predicted distribution over outcomes diverges from that anticipated under a steady state (*C*). This turns out to have the same form as KL-control schemes from engineering [9,10]:

(6) $$\begin{array}{cc} V(\pi ) & =-D_{KL}\left[P(o|\pi )||P(o|C)\right]\\ & =H\left[P(o|\pi )\right]+E_{P(o|\pi )}\left[\ln P(o|C)\right] \end{array}$$

The first line of Equation (6) says that the value (V) associated with a policy is the negative of the KL-Divergence between the distribution of outcomes under policies and under a desired (or steady state) distribution. The second line unpacks this in terms of a posterior predictive entropy and the expected log probability under the preferred distribution. Intuitively, the first term says that if everything is equally preferred, we should seek out those observations about which we are most uncertain. The second term is interpretable as a utility or expected reward function. From this perspective, the reward function is the log probability (plus or minus some additive constant). This suggests a bidirectional translation between probabilistic and reward-driven conceptions of behaviour.

### 4.2. Prefrontal Cortex

If we think about the kinds of paradigm common in animal and human research, the expression above is often enough to characterise behaviour. As an example, we consider the consequences of prefrontal cortical lesions and the ways in which these may be unpacked in terms of the above. The reasons for choosing this brain area are threefold. First, it has been consistently associated with decision-making and planning [62]. Second, there are dissociable phenotypes of prefrontal dysfunction [63]. Third, many animal experiments make use of gustatory stimuli (e.g., fruit juice) as “rewards” to motivate particular kinds of behaviour in response to auditory or visual cues [64]. The prefrontal cortex is uniquely placed to synthesise exteroceptive and interoceptive data to direct behaviour [65,66,67,68]. The relevance of this is that one of the most obvious settings in which Equation (6) applies is homeostasis [56]. This depends upon interoceptive data, which must be kept within tight bounds by the autonomic nervous system. In other words, the steady state density for interoceptive data is very precise (i.e., has low variance) [69,70,71]. Generalising homeostasis to allostasis [72,73], decisions made based upon external stimuli must also be motivated by ensuring interoceptive data fall within prescribed ranges [74].

To make this more concrete, it is worth thinking about the kinds of experimental setting used in studying prefrontal function. Paradigms include oculomotor delay-period tasks in primates [64] or contextual decision making in rodent tasks [75]. Common to these is that an exteroceptive stimulus is presented that indicates a task context. This might be visual, indicating the target location for a future saccade, or could be an auditory tone, indicating which lever to press. This is typically followed by a delay-period, in which the inferred context must be retained [76,77,78]. Finally, a choice must be made and communicated (e.g., through performing a saccade to a target location or pressing the appropriate level). Under each context, the choice has different consequences. If the chosen saccade location or level corresponds to the “correct” choice under the experimental context, the animal is rewarded. The reward often takes the form of fruit juice or milk, which have desirable interoceptive consequences.

Framed as a generative model [79], this says that we make use of hidden states comprising the task context and choice to predict outcomes that include exteroceptive and interoceptive stimuli. Exteroceptive stimuli depend only upon the task context, while the task context and choice conspire to predict the interoceptive input—as the juice is only received following the correct saccade. A prior belief that the juice will be obtained (in the *C*-distribution) ensures that policies leading to a high probability of juice are scored as more probable. Inverting this model means beliefs about context are inferred on the basis of exteroceptive data and act to contextualise the mapping from beliefs about choices to their interoceptive consequences [80]. The distinction between the two sets of beliefs is consistent with prefrontal connectivity, with medial prefrontal cortices highly connected to interoceptive regions (including the amygdala and insula cortex) [81,82,83,84] and lateral prefrontal cortices connected to exteroceptive cortices [85,86]—including via the superior longitudinal fasciculus discussed in Section 3.

The consequence of this is that lesions to different parts of the prefrontal cortex are accompanied by dissociable behavioural phenotypes. This offers a simple way of thinking about the distinction between medial and lateral prefrontal syndromes [79]. The formulation here suggests that lateral lesions should impair the ability to infer (and retain) beliefs about the experimental context—thereby precluding value-seeking behaviour, as the association between choices and their anticipated consequences are inappropriately contextualised [63]. In contrast, medial lesions are more likely to directly impair predictions about the interoceptive consequences of choice behaviour [87,88]. Despite normal intelligence and ability to contextualise which choice coheres with the context, such patients are apathetic and lack the motivation to select the “correct” answer [89,90,91]. The disconnection of desirable interoceptive consequences from beliefs about choices could underwrite the “insensitivity to future consequences” [92] associated with such patients.

## 5. Planning as Inference

### 5.1. Expected Free Energy

In Section 3 and Section 4, we saw that it is possible to score alternative behavioural policies in terms of their expected information gain (ℐ) or the divergence between anticipated and preferred outcomes (V). In this section, we formulate the two in terms of a single quantity, known as expected free energy (G) [25]. This is defined as follows:

(7) $$\begin{array}{cc} G(\pi ) & =H\left[P(o|\pi )\right]+E_{P(o|\pi )}\left[\ln P(o|C)\right]-E_{P(s|\pi )}\left[H\left[P(o|s,\pi )\right]\right]\\ & =ℐ(\pi )+E_{P(o|\pi )}\left[\ln P(o|C)\right]\\ & =V(\pi )-E_{P(s|\pi )}\left[H\left[P(o|s,\pi )\right]\right] \end{array}$$

Equation (7) makes the point that maximising the expected free energy favours both information-seeking and goal directed behaviours [93]. The second and third lines show how the measures of the preceding sections may be carved out of this single quantity. The key observation is that Equations (5) and (6) both include the predictive entropy. The implication here is that exploration and exploitation are *overlapping* as opposed to additive imperatives. Framing this in terms of a prior belief, we can use the softmax functional to convert these log probability measures into a normalised probability distribution. From this, we can also express the form of the posterior probability over policies:

(8) $$\begin{array}{cc} P(\pi ) & =\sigma_{\pi }\left[G(\pi )\right]\\ & \Rightarrow \ln Q(\pi )=G(\pi )+\ln P(o_{<}|\pi )+const.\\ & \Rightarrow Q(\pi )=\sigma_{\pi }\left[G(\pi )+\ln P(o_{<}|\pi )\right] \end{array}$$

The conditional probability of observations given policies (i.e., the marginal likelihood associated with a policy) holds the key as to why G is referred to as “expected free energy”. The reason for this name comes from its similarity with a quantity in Bayesian statistics known as “variational free energy” (ℱ) [94,95]. The similarity between the two is clearest in the following arrangements:

(9) $$\begin{array}{c} G(\pi )=E_{P(o|\pi )}\left[\ln P(o|C)\right]+E_{P(o,s|\pi )}\left[\ln P(s|o,\pi )\;\;-\ln P(s|\pi )\right]\\ ℱ(\pi )=\;\;\;\;\;\;\;\;\;\;\;\ln P(o_{<}|\pi )\;+\;\;\underset{=0}{\underset{︸}{E_{Q(s|\pi )}\left[\ln P(s|o_{<},\pi )-\ln Q(s|\pi )\right]}} \end{array}$$

While the form is similar, it is important to note their differences. The most prominent is that variational free energy is a function of (past) observations, while expected free energy averages over the (future) observations expected under a given policy. Variational free energy is an important quantity in Bayesian statistics as its maximisation (through changing *Q*) yields approximations to the posterior probability and to the marginal likelihood. In Section 2, we defined *Q* to be equal to the exact posterior. However, this need not be the case in general. Variational inference rests upon tractable parameterisations of this distribution that approximate the posterior, without having to be exact. Specifically:

(10) $$\begin{array}{c} P(s|o_{<},\pi )=\underset{Q(s|\pi )}{\arg\max}\;ℱ(\pi )\\ Q(s|\pi )=P(s|o_{<},\pi )\\ \;\;\;\;\Rightarrow ℱ(\pi )=\ln P(o_{<}|\pi )\\ \;\;\;\;\Rightarrow P(\pi |o_{<})=\sigma_{\pi }\left[ℱ(\pi )+G(\pi )\right] \end{array}$$

The inclusion of ℱ in this expression, accounting for observations that have already been made, provides us with an additional reason we might infer a course of action. This is that our sensory data tell us we are already doing so. In summary, this subsection highlights free energy and expected free energy as important quantities for motivated behaviour. The former is a measure of the consistency of a hypothesis (here—about how to act) with observed data. It is used to optimise beliefs relative to data. The latter turns this on its head and optimises data relative to beliefs. It generalises the discussion of information-seeking (explorative) behaviour from Section 3 and goal-directed (exploitative) behaviour from Section 4 under the same umbrella. 

### 5.2. Perseveration

**Figure 3:**
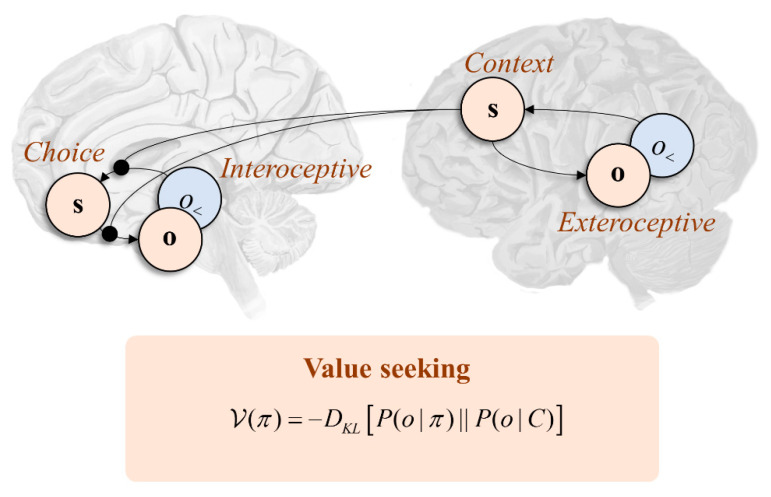
This schematic deals with the anatomy that underwrites many common experiments of prefrontal cortical function. This is formulated in line with lateral to medial functional gradients such that lateral cortices receive sensory information derived from exteroceptive sensory streams (e.g., visual and auditory cortices [85,86]), while medial and orbitofrontal regions receive input from interoceptive streams (e.g., the insula and amygdala [81,82,83,84]). This medial-to-lateral interoceptive-to-exteroceptive axis has been characterised as the “hot” and “cold” axis [98]. The relevance of this is that inferences about the context afforded by the external world influence the relationship between alternative choices a creature might make (policy-dependent states) and the interoceptive consequences of those choices. For example, an experimental stimulus could provide a clue as to which option results in a gustatorily rewarding outcome. As we said above, interoceptive outcomes are the most tightly regulated. This implicates medial regions like the orbitofrontal cortex in this aspect of motivated behaviour.

At this point, it is worth unpacking an interesting consequence of Equation (10). This is that it implies a certain sort of behavioural momentum. Once a policy has started to be enacted, the evidence attained in favour of that policy promotes its continuation. This provides an interesting perspective on a common consequence of frontal lobe damage. Patients with lesions in their frontal cortex often exhibit perseveration [96,97], or the repetition of a phrase or gesture that has ceased to be appropriate. Equation (10) hints at why this might be. If frontal lesions impair the computation of the expected free energy—for example, by impairing the prediction of likely outcomes as in Figure 3—Equation (10) becomes dominated by the variational free energy, which supports any policy that has already been initiated.

As an example, imagine asking someone to copy what you do and clapping three times. While the instruction might be sufficient to initiate the behaviour, a patient who is less able to predict the consequences of their behaviour in relation to desired outcomes will be unable to maintain the differentiation of alternative policies based upon the expected free energy. They will end up relying upon the variational free energy term instead. As visual, auditory, and proprioceptive data provide evidence for “clapping”, they may continue to clap even once they have completed the required three. Regardless of the choice of prior beliefs for a policy, it is interesting that perseverative behaviour is an almost inevitable consequence of planning as inference.

## 6. Deep Generative Models

### 6.1. Temporal Hierarchies

**Figure 4:**
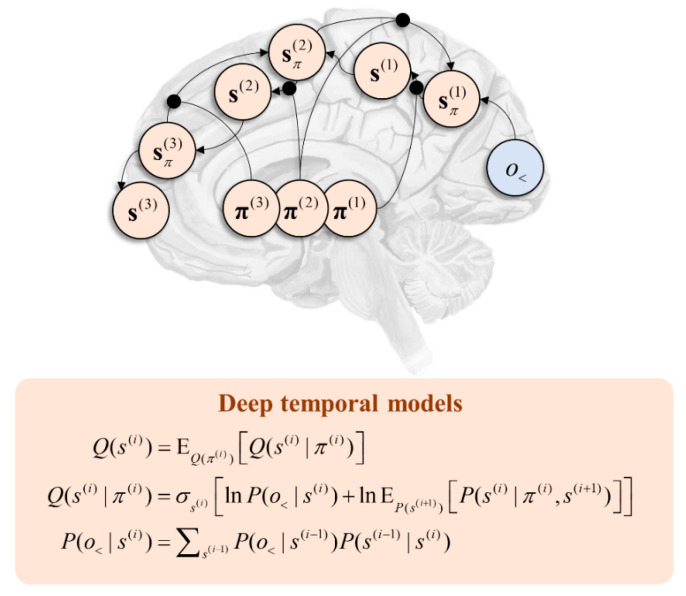
This graphic shows the form of message passing implied by a deep temporal model. This image focuses upon inference about states but emphasises the importance of inferences about policies in contextualising message passing between hierarchical levels. Each level is indexed with a bracketed superscript. At each level, ascending messages from the level below constrain activity based upon sensory data (either vicariously or directly—in the case of level 1). This enables inferences about the states conditioned upon a policy. This may be averaged under beliefs about the policy at that level. The resulting inference about states provides the ascending message to the higher level. Inferences about states conditioned upon a policy form descending messages, averaged under beliefs about the policy at the same level. Note the asymmetry of message passing—characteristic of cortical connectivity [104,105]—in the ascending and descending directions as a result of this averaging.

The generative models outlined above may be generalised to account for variables that evolve over multiple timescales. A canonical example of this is language, as we decompose speech or writing into very fast units (phonemes or letters) that are predicted by slower units (words), themselves predicted by slower (sentences), slower (narratives), and slower (stories) units [99]. This deep structure may be formalised through a generative model by repeating the structure we have seen in Figure 1 such that lower-level states act as if they were observations from the perspective of high-level states. Figure 4 illustrates the message passing associated with this sort of hierarchy. Note the similarity with Figure 2 but the repetition of that motif in a hierarchical structure. Evidence in favour of such temporal hierarchies in the brain is abundant [100,101,102,103].

Temporal hierarchy offers two additional constraints on behaviour that we must consider in a general setting. The first is that the steady state distribution may be local in time. Over a very fast timescale, it is approximately ergodic, but it may change gradually over a slower timescale. This implies the relationship between *C* and *o* must be contextualised by the higher level. Similarly, prior experience may have found some experimental designs more fitting in some contexts than others. More informally, creatures develop habits [106]. To account for this, we can include an additional term (E) that provides this contextualisation and is a function of slowly evolving states. This is termed an *empirical* prior and contributes to the prior in Equation (8) in an analogous manner to the expected free energy. Empirical priors arise in hierarchical models where more abstract inferences (e.g., “I am in a car”) act as prior beliefs for more concrete inferences over faster timescales (e.g., “I am driving”). There is a sense in which this plays the role of a state-action policy of the sort found in reinforcement learning schemes [107], as it specifies what to do conditioned upon a (slowly evolving) state. Heuristically, the relationship between E and G is a point of connection with the relationship between “model-free” and “model-based” systems [108] but, formulated this way, commits to the idea that they are both explicitly model-based. Another perspective is that empirical priors mediating a top-down control over behaviour represent a form of cognitive control [109].

Because they share the posterior predictive entropy, it makes little sense to carve up the ℐ and V terms introduced in Section 3 and Section 4, and combined in Section 5. However, there is a natural separation between these and E. This lets us differentially weight this context-dependent term against the expected free energy, using a “precision” or inverse temperature (where temperature quantifies dispersion or variance) parameter, *β*:

(11) $$\begin{array}{l} P(\pi^{\left(i\right)})=E_{P(\beta^{\left(i\right)})}\left[P(\pi^{\left(i\right)}|\beta^{\left(i\right)})\right]\\ Q(\pi^{\left(i\right)})=E_{Q(\beta^{\left(i\right)})}\left[Q(\pi^{\left(i\right)}|\beta^{\left(i\right)})\right]\\ P(\pi^{\left(i\right)}|\beta^{\left(i\right)})=E_{P(s^{\left(i+1\right)})}\left[\sigma_{\pi^{\left(i\right)}}\left[\beta^{\left(i\right)}E^{\left(i\right)}(\pi^{\left(i\right)},s^{\left(i+1\right)})+G^{\left(i\right)}(\pi^{\left(i\right)},s^{\left(i+1\right)})\right]\right]\\ Q(\pi^{\left(i\right)}|\beta^{\left(i\right)})=E_{Q(s^{\left(i+1\right)})}\left[\sigma_{\pi^{\left(i\right)}}\left[\beta^{\left(i\right)}E(\pi^{\left(i\right)},s^{\left(i+1\right)})+{ℱ}^{\left(i\right)}(\pi^{\left(i\right)},s^{\left(i+1\right)})+G^{\left(i\right)}(\pi^{\left(i\right)},s^{\left(i+1\right)})\right]\right] \end{array}$$

Interpreting these equations, we see that posterior beliefs about the policy depend upon an average (under posterior beliefs about the precision) of the conditional probability of the policy given the precision and the data [25]. This is shown in the second line. This suggests a modulatory influence of the precision that, referring to the fourth line, weights the balance between the empirical prior relative to ℱ and G. The empirical prior depends upon higher level states directly, while the free energies depend upon these only via beliefs about states at the same level.

### 6.2. Direct and Indirect Pathways

Equation (11) may be regarded as a relatively general expression of behavioural motivation. It accounts for the influence of slowly changing contexts of the sort that can form habits, for a behavioural momentum that entails the continuation of current policies and maximisation of the expected free energy. The last of these incorporates both exploitative and explorative motivations. How do we now interpret this prior in terms of its neurobiological substrates? The simplest way of doing this is to think about the consequences of changing the free parameter (*β*) and what we might expect to happen.

When *β* is very large, inference about policies is tightly constrained by inferences at the slower timescale. The temporal coarseness of this influence implies that its effects must be relatively non-specific. As opposed to selecting a specific policy, the E potential emphasised by *β* effectively acts to make all context-inappropriate policies implausible. As such, we can think of this potential as contributing to inference about what not to do. Those policies unlikely to be suppressed by this term include those that are common in nearly all contexts—for example, the maintenance of postural tone. In short, a very large *β* predicts staying still. In contrast, when *β* is very small, the expected free energy term dominates Equation (11). This implies the promotion of behavioural policies, even when the slower timescale suggests they are inappropriate. In other words, behaviour may appear impulsive.

This raises the question as to where in the brain we find a variable that at one extreme leads to the cessation of behaviour and at the other leads to its promotion. The obvious candidate here is striatal dopamine. Its depletion in severe Parkinson’s disease leads to akinesia while exogenous dopamine agonists promote impulsive behaviours, such as gambling [110,111,112]. The physiological role of this substance is to modulate the balance between the “direct” and “indirect” pathways through a set of subcortical structures known as the basal ganglia [113]. The former is involved in behavioural promotion and the latter in suppression. This has been demonstrated through optogenetic manipulations [114] that target either the D1-receptor expressing medium spiny neurons (MSNs) of the direct pathway or the D2-receptor expressing MSNs of the indirect pathway. The optogenetic activation of these neurons in rodents induces the associated behavioural phenotypes (activity versus freezing). Framing this in terms of planning as inference, the direct pathway infers what to do, while the indirect infers what not to. This implies dopamine may act as the inverse of the *β* parameter.

**Figure 5:**
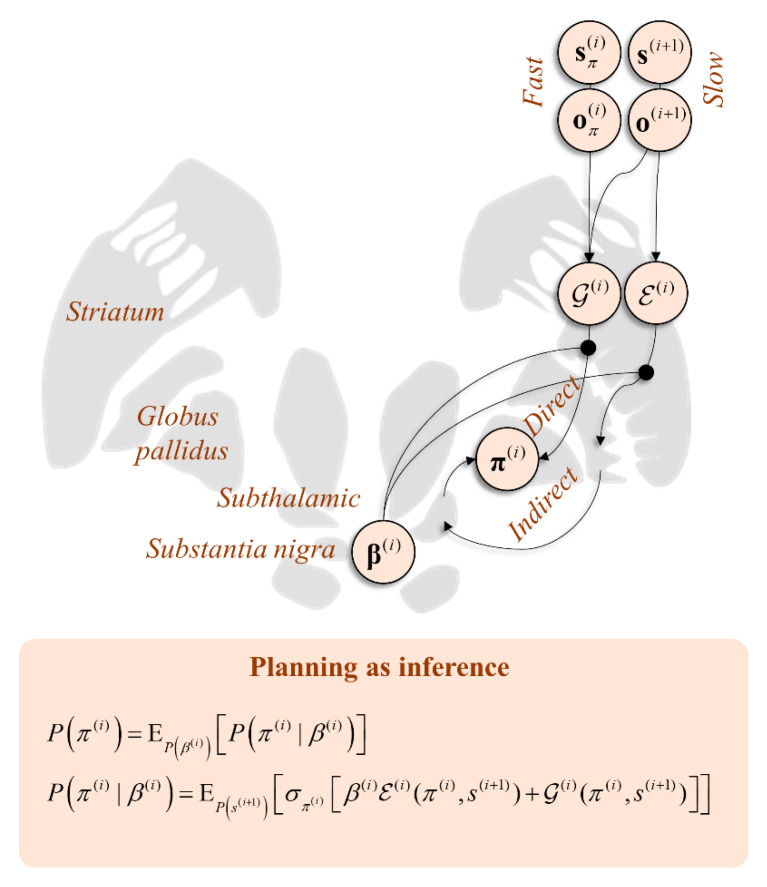
This coronal section through the basal ganglia shows how inferences over different timescales interact in informing policy priors. Here, the distinction between the E and G potentials has been cartooned as the distinction between the direct and indirect pathways, the softmax parameter *β* influencing the balance between the two. Note that the softmax ensures a form of reciprocal inhibition, such that a large weight on E implies a diminishing effect of G. This is the reason we show the softmax parameter as influencing both the direct and indirect pathways. By facilitating one, the other is implicitly suppressed.

Figure 5 shows a schematic coronal section through the basal ganglia, with the message passing implied by Equation (11) mapped to the direct and indirect pathways. This highlights the consistency with conceptual models of basal ganglia function. For example [115], it has been suggested that the direct pathway mediates a fast and focused inhibition of the globus pallidus internus, followed by a broader and slower excitation. These cause the excitation and inhibition of the targets of the globus pallidus, respectively. This is thought to ensure a “centre-surround” pattern that facilitates only the appropriate motor programmes. This is consistent with the fast processes computing the expected free energy facilitating action, with the broader contextualisation of the slower pathway mediating empirical priors. Comparing with Figure 4, the anatomy of Figure 5 predicts the involvement of the temporally slower (i.e., frontal) regions in targeting indirect pathway neurons, but both fast and slow influences over the direct pathway. This is consistent with the anatomical distribution of cortical inputs to the basal ganglia [116] and with the morphology of D1- and D2-expressing MSNs, the former exhibiting larger dendritic arbours [117]. As such, the anatomy of Figure 5 is endorsed by evidence from optogenetics, clinical pathology (e.g., Parkinsonism), and cellular morphology.

## 7. Precision Optimisation

### 7.1. Reciprocal Messages

In the above, we made use of a posterior distribution over the precision term. This acts to balance behavioural promotion or suppression, so is central to the theme of this article. As such, it is worth thinking about where this posterior comes from. Like any other parameter in the models dealt with here, this may be optimised. The form of the posterior depends upon beliefs about policies and on priors over the precision:

(12) $$\begin{array}{l} Q(\beta^{\left(i\right)})=E_{Q(\pi^{\left(i\right)})}\left[Q(\beta^{\left(i\right)}|\pi^{\left(i\right)})\right]\\ Q(\beta^{\left(i\right)}|\pi^{\left(i\right)})=\sigma_{\beta^{\left(i\right)}}\left[\ln Q(\pi^{\left(i\right)}|\beta^{\left(i\right)})+\ln P(\beta^{\left(i\right)})\right] \end{array}$$

The simplicity of this expression rests upon the fact that the precision only influences data via the policies. The interesting thing about Equation (12) is that it emphasises the reciprocity of Bayesian message passing. It implies that the modulatory influence of precision on policies is complemented by a modulatory influence of (beliefs about) policies on beliefs about precision. By inspection of Equations (11) and (12), we see that large values of *β* are more probable (*a posteriori*) when the free energy is more consistent with the empirical prior than with the expected free energy. As highlighted above, the empirical prior, determined by coarse-grained contextual information, is often less precise than the distribution under the expected free energy alone. The implication is that the precision of the empirical prior will drop as confidence in the policy increases and the expected free energy dominates. Under the dopaminergic theory of precision outlined in the previous section, this implies greater confidence in “how I am acting”, due to either an increase in potential information gain or extrinsic value, leads to a drop in empirical prior precision and an increase in dopaminergic signalling. This is exactly what is found [118] but often interpreted in terms of “reward prediction error” [119] perhaps due to the prevalence of experimental designs that use extrinsic value (i.e., rewards) to motivate behavioural compliance.

### 7.2. Nigrostriatal Loops

**Figure 6:**
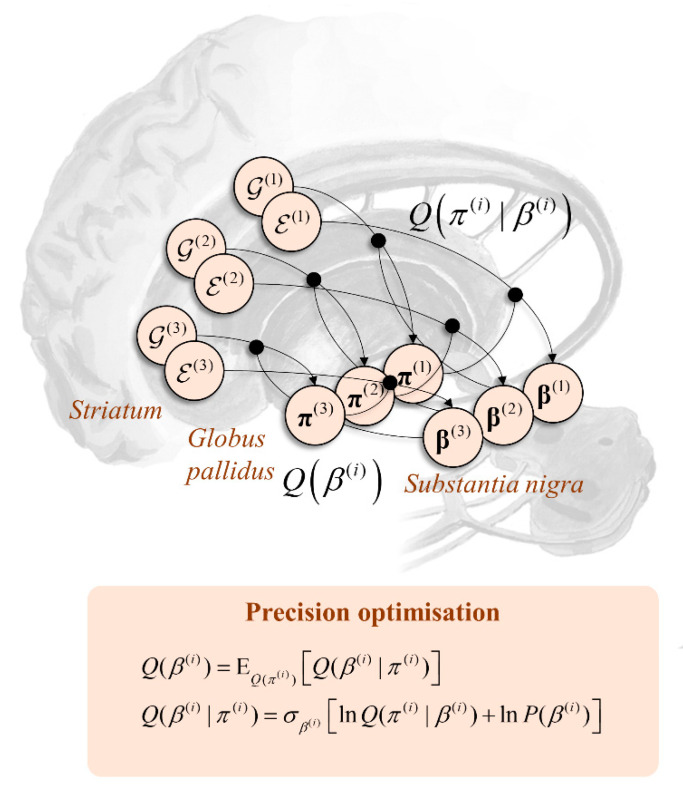
This graphic emphasises the reciprocity of Bayesian message passing. The structure from Figure 5 has been incorporated and extended to deal with inference about the precision. Here, the influence of the precision over the mapping from the expected free energy and empirical priors to the policies is reciprocated, such that the precision is itself updated based upon an expectation under posterior beliefs about the policy. This is depicted here in terms of a series of hierarchical nigrostriatal loops, each of which deals with a separate timescale of inference.

As indicated above, the influence of beliefs about precision on beliefs about policies implies reciprocation. This is interesting from a neuroanatomical standpoint as, under the anatomy of Figure 5, it mandates connectivity loops between the striatum and substantia nigra, as depicted in Figure 6. This is a characteristic feature of nigrostriatal organisation [120], with multiple hierarchically arranged loops. While this is a distributed network whose components evolve in parallel, it is sometimes useful to decompose it as if it were sequential to aid intuition. Here, the narrative starts with the cortical input to the striatal MSNs, which is used to compute the expected free energy and empirical priors over policies. Along the axis of the striatum, these calculations happen in parallel at each level of the temporal hierarchy. Beliefs about the policies based upon these quantities depend upon the precision (*β*). Messages passed from the striatum to the globus pallidus internus—both directly and indirectly—must therefore be averaged under current beliefs about the precision. This manifests as a dopaminergic modulation of the balance between the two pathways. Once beliefs about policies are updated to their posteriors (in the globus pallidus), these are used to modulate projections from the striatum directly to the substantia nigra (pars compacta), updating beliefs about the precision and completing the loop.

A final point to add to this is that the priors over precision may themselves be context dependent [121]. This implies *β*^(*i*)^ sits in the Markov blanket of *s*^(*i*+1)^ and vice versa. As such, associating the former with the dopaminergic midbrain and the latter with frontal cortical regions implies reciprocal connectivity between the two. Interestingly, the presence of axonal terminals originating in the ventral tegmental area has been used as a means of defining the prefrontal cortex [122]. This connectivity may be seen as evidence in favour of the brain’s use of a generative model that conditions policy-precision on higher level states.

## 8. Discussion

We have formalised a series of hypotheses for why someone might engage in a particular behaviour. These hypotheses may be categorised into four groups, some of which may be subdivided further. The first is curiosity. Curiosity can be broken down into curiosity about the dependencies between things (novelty seeking) or about the things themselves (salience attribution) [123]. Each of these may be further subdivided into seeking hard-to-predict sensory data (i.e., maximising a predictive entropy) and an aversion to ambiguity. The second reason for doing something is that it may be motivated by desired or anticipated sensations. We saw that this overlaps with information-seeking behaviour. The third reason someone engages in a behaviour is that they have already started doing so, and the resulting sensory data reinforces their inference that this is what they are doing. Finally, behaviour may be driven by inferences at slower timescales that specify a range of plausible (and implausible) options given the context in which someone finds themself.

It is important to outline these alternatives explicitly, as they form a hypothesis space for behavioural sciences. Their mathematical formalisation enables explicit comparison of the evidence each affords for a given behaviour. For example, Mirza et al. [38] compared the evidence for models that included or excluded the information-seeking terms (Equation (3)) in the expected free energy (Equation (7)) in relation to data collected in a simple behavioural task. This demonstrated the superiority of explanations of behaviour that appeal to curiosity. Similar comparisons, for any given behaviour, may be made to assess the contribution of any of the terms above.

This article has focused upon the biological substrates of these priors. By unpacking the conditional dependencies in the implicit generative model, we saw that the connectivity structures that emerge may be associated with neuroanatomical constructs. Understanding the computational anatomy of motivation is vital in studying behavioural disorders or variants in a biologically grounded way. As an example of the utility of this approach, we briefly (and superficially) highlight its application in understanding behavioural variations in personality disorders from a functional biological perspective. Personality disorders such as antisocial personality disorder or “psychopathy” are not typically thought of in terms of their underlying biology. When they are, the focus tends to be at the genetic or molecular level [124].

The computational anatomy outlined above lets us pose hypotheses at a systems level, going from structure to behaviour. In the context of psychopathy, the expression of behavioural traits including remorselessness and grandiosity, in terms of a formal model, facilitated clear links between behaviour and neuroanatomical variations [125]. Both traits may be thought of in terms of beliefs about self-worth, associated with the medial and orbitofrontal prefrontal cortices (as evidenced by neuropsychological lesion studies and neuroimaging [126,127]). Drawing from the account of medial prefrontal inference outlined in Section 4, a plausible internal model for self-worth is that it predicts data related to the approval of others—that we might expect to be associated with structures like the amygdala (consistent with functional neuroimaging measurements of responses to stimuli that are or are not socially informative [128])—over which we have preferences. In addition, self-worth is policy dependent. Behaving charitably increases our sense of worth, while it is diminished by behaving with cruelty or indifference. This appeals to the depiction of beliefs averaged under policies in Figure 4 and the associated cortico-striatal loops.

Once we have this model in mind, we see how the loss of white matter integrity in the uncinate fasciculus—connecting the orbitofrontal cortex to the amygdala—in psychopathy [129] could lead to a diminished remorsefulness in the face of social disapproval. This is like a selective form of the apathy in medial prefrontal syndromes outlined in Section 3. When this is combined with the disruption of prefrontal cortico-striatal (functional or structural) connectivity [130,131], people become resistant to the attenuation of inferred self-worth normally induced by engaging in antisocial behaviours. This offers an explanation for grandiosity. The example of psychopathy is a useful illustration of how “planning as inference” takes us past a purely descriptive account of unusual behaviour. It lets us formulate neurobiological theories.

## 9. Conclusions

In this paper, we have outlined the formalism of planning as inference—as expressed under active inference. This amounts to specifying motivation in terms of contributions to a prior belief about what to do next. Following Bayesian inference, posterior beliefs about how to act comprise contributions from information and value-seeking objectives, supplemented with a behavioural momentum that promotes the continuation of a policy that has been started. These all promote activity but are balanced by empirical priors that rule out those policies that are inappropriate in a given context. This balance between doing and not doing is modulated by a precision parameter. Such modulatory influences are highly consistent with the modulation of direct and indirect basal ganglia pathways by dopamine. Ultimately, we may interpret the role of this substance as weighting inferences about what to do against those about what not to.

## References

[1] Attias H. Planning by Probabilistic Inference. Proceedings of the 9th International Workshop on Artificial Intelligence and Statistics.

[2] Botvinick M., Toussaint M. (2012). Planning as inference. Trends Cogn. Sci..

[3] Friston K., Samothrakis S., Montague R. (2012). Active inference and agency: Optimal control without cost functions. Biol. Cybern..

[4] Berger-Tal O., Nathan J., Meron E., Saltz D. (2014). The Exploration-Exploitation Dilemma: A Multidisciplinary Framework. PLoS ONE.

[5] Lindley D.V. (1956). On a Measure of the Information Provided by an Experiment. Ann. Math. Statist..

[6] Itti L., Baldi P. (2006). Bayesian surprise attracts human attention. Adv. Neural Inf. Process. Syst..

[7] Oudeyer P.-Y., Kaplan F. (2007). What is Intrinsic Motivation? A Typology of Computational Approaches. Front. Neurorobot..

[8] Biehl M., Guckelsberger C., Salge C., Smith S.C., Polani D. (2018). Expanding the Active Inference Landscape: More Intrinsic Motivations in the Perception-Action Loop. Front. Neurorobot..

[9] Todorov E. Linearly-solvable Markov decision problems. Proceedings of the Advances in Neural Information Processing Systems.

[10] Kinjo K., Uchibe E., Doya K. (2013). Evaluation of linearly solvable Markov decision process with dynamic model learning in a mobile robot navigation task. Front. Neurorobot..

[11] Parr T., Markovic D., Kiebel S.J., Friston K.J. (2019). Neuronal message passing using Mean-field, Bethe, and Marginal approximations. Sci. Rep..

[12] Parr T., Friston K.J. (2018). The Anatomy of Inference: Generative Models and Brain Structure. Front. Comput. Neurosci..

[13] Jahanshahi M., Obeso I., Rothwell J.C., Obeso J.A. (2015). A fronto-striato-subthalamic-pallidal network for goal-directed and habitual inhibition. Nat. Rev. Neurosci..

[14] Gurney K., Prescott T.J., Redgrave P. (2001). A computational model of action selection in the basal ganglia. I. A new functional anatomy. Biol. Cybern..

[15] Fuster J.M., Bodner M., Kroger J.K. (2000). Cross-modal and cross-temporal association in neurons of frontal cortex. Nature.

[16] van de Laar T., de Vries B. (2016). A Probabilistic Modeling Approach to Hearing Loss Compensation. IEEE/ACM Trans. Audio Speech Lang. Process..

[17] Forney G.D., Vontobel P.O. (2011). Partition functions of normal factor graphs. arXiv.

[18] Dauwels J. On variational message passing on factor graphs. Proceedings of the ISIT 2007 IEEE International Symposium on Information Theory.

[19] Loeliger H.A. (2004). An introduction to factor graphs. IEEE Signal Process. Mag..

[20] de Vries B., Friston K.J. (2017). A Factor Graph Description of Deep Temporal Active Inference. Front. Comput. Neurosci..

[21] Loeliger H.A., Dauwels J., Hu J., Korl S., Ping L., Kschischang F.R. (2007). The factor graph approach to model-based signal processing. Proc. IEEE.

[22] Hohwy J. (2016). The Self-Evidencing Brain. Noûs.

[23] Hohwy J. (2020). Self-supervision, normativity and the free energy principle. Synthese.

[24] van de Laar T.W., de Vries B. (2019). Simulating Active Inference Processes by Message Passing. Front. Robot. AI.

[25] Friston K., FitzGerald T., Rigoli F., Schwartenbeck P., Pezzulo G. (2017). Active Inference: A Process. Theory. Neural Comput..

[26] Hebb D.O. (1949). The first stage of perception: Growth of the assembly. Organ. Behav..

[27] Brown T.H., Zhao Y., Leung V., Squire L.R. (2009). Hebbian Plasticity A2. Encyclopedia of Neuroscience.

[28] George D., Hawkins J. (2009). Towards a mathematical theory of cortical micro-circuits. PLoS Comput. Biol..

[29] Pearl J. (1988). Probabilistic Reasoning in Intelligent Systems: Networks of Plausible Inference.

[30] Clark A. (2018). A nice surprise? Predictive processing and the active pursuit of novelty. Phenomenol. Cogn. Sci..

[31] MacKay D.J. (1992). Information-based objective functions for active data selection. Neural Comput..

[32] Itti L., Koch C. (2000). A saliency-based search mechanism for overt and covert shifts of visual attention. Vis. Res..

[33] Mirza M.B., Adams R.A., Mathys C.D., Friston K.J. (2016). Scene Construction, Visual Foraging, and Active Inference. Front. Comput. Neurosci..

[34] Andreopoulos A., Tsotsos J. (2013). A computational learning theory of active object recognition under uncertainty. Int. J. Comput. Vis..

[35] Ognibene D., Baldassarre G. (2014). Ecological Active Vision: Four Bio-Inspired Principles to Integrate Bottom-Up and Adaptive Top-Down Attention Tested with a Simple Camera-Arm Robot. IEEE Trans. Auton. Ment. Dev..

[36] Wurtz R.H., McAlonan K., Cavanaugh J., Berman R.A. (2011). Thalamic pathways for active vision. Trends Cogn. Sci..

[37] Friston K., Adams R.A., Perrinet L., Breakspear M. (2012). Perceptions as Hypotheses: Saccades as Experiments. Front.Psychol..

[38] Mirza M.B., Adams R.A., Mathys C., Friston K.J. (2018). Human visual exploration reduces uncertainty about the sensed world. PLoS ONE.

[39] Yang S.C., Lengyel M., Wolpert D.M. (2016). Active sensing in the categorization of visual patterns. eLife.

[40] Halligan P.W., Marshall J.C. (1998). Neglect of Awareness. Conscious. Cogn..

[41] Albert M.L. (1973). A simple test of visual neglect. Neurology.

[42] Fullerton K.J., McSherry D., Stout R.W. (1986). Albert’s Test: A Neglected Test. of Perceptual Neglect. Lancet.

[43] Husain M., Mannan S., Hodgson T., Wojciulik E., Driver J., Kennard C. (2001). Impaired spatial working memory across saccades contributes to abnormal search in parietal neglect. Brain.

[44] Malhotra P., Mannan S., Driver J., Husain M. (2004). Impaired Spatial Working Memory: One Component of the Visual Neglect Syndrome?. Cortex.

[45] Mannan S.K., Mort D.J., Hodgson T.L., Driver J. (2005). Revisiting Previously Searched Locations in Visual Neglect: Role of Right Parietal and Frontal Lesions in Misjudging Old Locations as New. J. Cogn. Neurosci..

[46] Corbetta M., Shulman G.L. (2002). Control of goal-directed and stimulus-driven attention in the brain. Nat. Rev. Neurosci..

[47] Bartolomeo P., Thiebaut de Schotten M., Chica A.B. (2012). Brain networks of visuospatial attention and their disruption in visual neglect. Front. Hum. Neurosci..

[48] Makris N., Kennedy D.N., McInerney S., Sorensen A.G., Wang R., Caviness V.S., Pandya D.N. (2004). Segmentation of Subcomponents within the Superior Longitudinal Fascicle in Humans: A Quantitative, In Vivo, DT-MRI Study. Cereb. Cortex.

[49] Shah A., Goel A., Jhawar S.S., Patil A., Rangnekar R., Goel A. (2019). Neural Circuitry: Architecture and Function—A Fiber Dissection Study. World Neurosurg..

[50] de Schotten M.T., Dell’Acqua F., Forkel S., Simmons A., Vergani F., Murphy D.G.M., Catani M. (2011). A lateralized brain network for visuospatial attention. Nat. Neurosci..

[51] Zimmermann E., Lappe M. (2016). Visual Space Constructed by Saccade Motor Maps. Front. Hum. Neurosci..

[52] Parr T., Mirza M.B., Cagnan H., Friston K.J. (2019). Dynamic causal modelling of active vision. J. Neurosci..

[53] Parr T., Friston K.J. (2018). The Computational Anatomy of Visual Neglect. Cereb. Cortex.

[54] Parr T., Friston K.J., Hodgson T. (2019). Active Inference, Novelty and Neglect. Processes of Visuospatial Attention and Working Memory.

[55] Parr T., Friston K.J. (2017). Uncertainty, epistemics and active inference. J. R. Soc. Interface.

[56] Cannon W.B. (1929). Organization for physiological homeostasis. Physiol. Rev..

[57] Kwon C., Ao P., Thouless D.J. (2005). Structure of stochastic dynamics near fixed points. Proc. Natl. Acad. Sci. USA.

[58] Parr T., Costa L.D., Friston K. (2020). Markov blankets, information geometry and stochastic thermodynamics. Philos. Trans. Royal Soc. A Math. Phys. Eng. Sci..

[59] Friston K. (2019). A free energy principle for a particular physics. arXiv.

[60] Friston K., Ao P. (2012). Free Energy, Value, and Attractors. Comput. Math. Methods Med..

[61] Friston K. (2013). Life as we know it. J. R. Soc. Interface.

[62] Tanji J., Hoshi E. (2001). Behavioral planning in the prefrontal cortex. Curr. Opin. Neurobiol..

[63] Szczepanski S.M., Knight R.T. (2014). Insights into Human Behavior from Lesions to the Prefrontal Cortex. Neuron.

[64] Funahashi S. (2015). Functions of delay-period activity in the prefrontal cortex and mnemonic scotomas revisited. Front. Syst. Neurosci..

[65] Barbas H. (2015). General Cortical and Special Prefrontal Connections: Principles from Structure to Function. Annu. Rev. Neurosci..

[66] Barbas H., Zikopoulos B. (2007). The Prefrontal Cortex and Flexible Behavior. Neuroscientist.

[67] Price J.L., Carmichael S.T., Drevets W.C., Holstege G., Bandler R., Saper C.B. (1996). Networks related to the orbital and medial prefrontal cortex; a substrate for emotional behavior?. Progress in Brain Research.

[68] Nee D.E., D’Esposito M. (2016). The hierarchical organization of the lateral prefrontal cortex. eLife.

[69] Ondobaka S., Kilner J., Friston K. (2017). The role of interoceptive inference in theory of mind. Brain Cogn..

[70] Seth A.K., Friston K.J. (2016). Active interoceptive inference and the emotional brain. Philos. Trans. Royal Soc. B Biol. Sci..

[71] Seth A.K. (2013). Interoceptive inference, emotion, and the embodied self. Trends Cogn. Sci..

[72] Corcoran A.W., Hohwy J. (2017). Allostasis, interoception, and the free energy principle: Feeling our way forward. The Interoceptive Mind: From Homeostasis to Awareness.

[73] Corcoran A.W., Pezzulo G., Hohwy J. (2020). From allostatic agents to counterfactual cognisers: Active inference, biological regulation, and the origins of cognition. Biol. Philos..

[74] Allen M., Levy A., Parr T., Friston K.J. (2019). In the Body’s Eye: The Computational Anatomy of Interoceptive Inference. BioRxiv.

[75] Wimmer R.D., Schmitt L.I., Davidson T.J., Nakajima M., Deisseroth K., Halassa M.M. (2015). Thalamic control of sensory selection in divided attention. Nature.

[76] Goldman-Rakic P.S. (2011). Circuitry of Primate Prefrontal Cortex and Regulation of Behavior by Representational Memory. Comprehensive Physiology.

[77] Arnsten A.F.T. (2013). The Neurobiology of Thought: The Groundbreaking Discoveries of Patricia Goldman-Rakic 1937–2003. Cereb. Cortex.

[78] Coull J.T. (1998). Neural correlates of attention and arousal: Insights from electrophysiology, functional neuroimaging and psychopharmacology. Progress Neurobiol..

[79] Parr T., Rikhye R.V., Halassa M.M., Friston K.J. (2020). Prefrontal Computation as Active Inference. Cereb. Cortex.

[80] Damasio A.R. (1996). The somatic marker hypothesis and the possible functions of the prefrontal cortex. Philos. Trans. R. Soc. Lond. B.

[81] Barbas H., García-Cabezas M.Á. (2016). How the prefrontal executive got its stripes. Curr. Opin. Neurobiol..

[82] Gu X., Hof P.R., Friston K.J., Fan J. (2013). Anterior Insular Cortex and Emotional Awareness. J. Comp. Neurol..

[83] Mufson E.J., Mesulam M.M., Pandya D.N. (1981). Insular interconnections with the amygdala in the rhesus monkey. Neuroscience.

[84] Devinsky O., Morrell M.J., Vogt B.A. (1995). Contributions of anterior cingulate cortex to behaviour. Brain.

[85] Romanski L.M., Tian B., Fritz J., Mishkin M., Goldman-Rakicm P.S., Goldman-Rakic J.P. (1999). Dual streams of auditory afferents target multiple domains in the primate prefrontal cortex. Nat. Neurosci..

[86] Wilson F.A., Scalaidhe S.P., Goldman-Rakic P.S. (1993). Dissociation of object and spatial processing domains in primate prefrontal cortex. Science.

[87] O’Callaghan C., Vaghi M.M., Brummerloh B., Cardinal R.N., Robbins T.W. (2018). Impaired awareness of action-outcome contingency and causality during healthy ageing and following ventromedial prefrontal cortex lesions. Neuropsychologia.

[88] Harlow J.M. (1999). Passage of an Iron Rod Through the Head. J. Neuropsychiatry Clinical Neurosci..

[89] Eslinger P.J., Damasio A.R. (1985). Severe disturbance of higher cognition after bilateral frontal lobe ablation: Patient EVR. Neurology.

[90] Damasio H., Grabowski T., Frank R., Galaburda A.M., Damasio A.R. (1994). The return of Phineas Gage: Clues about the brain from the skull of a famous patient. Science.

[91] Papez J.W. (1937). A proposed mechanism of emotion. Arch. Neurol. Psychiatry.

[92] Bechara A., Damasio A.R., Damasio H., Anderson S.W. (1994). Insensitivity to future consequences following damage to human prefrontal cortex. Cognition.

[93] Friston K., Rigoli F., Ognibene D., Mathys C., Fitzgerald T., Pezzulo G. (2015). Active inference and epistemic value. Cogn. Neurosci..

[94] Dayan P., Hinton G.E., Neal R.M., Zemel R.S. (1995). The Helmholtz machine. Neural Comput..

[95] Beal M.J. (2003). Variational Algorithms for Approximate Bayesian Inference.

[96] Freedman M., Black S., Ebert P., Binns M. (1998). Orbitofrontal function, object alternation and perseveration. Cereb. Cortex.

[97] Nyhus E., Barceló F. (2009). The Wisconsin Card Sorting Test and the cognitive assessment of prefrontal executive functions: A critical update. Brain Cogn..

[98] O’Reilly R.C. (2010). The What and How of prefrontal cortical organization. Trends Neurosci..

[99] Friston K.J., Rosch R., Parr T., Price C., Bowman H. (2017). Deep temporal models and active inference. Neurosci. Biobehav. Rev..

[100] Kiebel S.J., Daunizeau J., Friston K.J. (2008). A Hierarchy of Time-Scales and the Brain. PLoS Comput. Biol..

[101] Cocchi L., Sale M.V., Gollo L.L., Bell P.T., Nguyen V.T., Zalesky A., Breakspear M., Mattingley J.B. (2016). A hierarchy of timescales explains distinct effects of local inhibition of primary visual cortex and frontal eye fields. Elife.

[102] Hasson U., Yang E., Vallines I., Heeger D.J., Rubin N. (2008). A Hierarchy of Temporal Receptive Windows in Human Cortex. J. Neurosci. Off. J. Soc. Neurosci..

[103] Murray J.D., Bernacchia A., Freedman D.J., Romo R., Wallis J.D., Cai X., Padoa-Schioppa C., Pasternak T., Seo H., Lee D. (2014). A hierarchy of intrinsic timescales across primate cortex. Nat. Neurosci..

[104] Zeki S., Shipp S. (1988). The functional logic of cortical connections. Nature.

[105] Felleman D.J., Van Essen D.C. (1991). Distributed Hierarchical Processing in the Primate Cerebral Cortex. Cereb. Cortex.

[106] FitzGerald T., Dolan R., Friston K. (2014). Model averaging, optimal inference, and habit formation. Front. Hum. Neurosci..

[107] Watkins C.J., Dayan P. (1992). Q-learning. Mach. Learn..

[108] Lee S.W., Shimojo S., O’Doherty J.P. (2014). Neural computations underlying arbitration between model-based and model-free learning. Neuron.

[109] Barceló F., Cooper P.S. (2018). Quantifying Contextual Information for Cognitive Control. Front. Psychol..

[110] Galea J.M., Bestmann S., Beigi M., Jahanshahi M., Rothwell J.C. (2012). Action Reprogramming in Parkinson’s Disease: Response to Prediction Error Is Modulated by Levels of Dopamine. J. Neurosci..

[111] Frank M.J. (2005). Dynamic Dopamine Modulation in the Basal Ganglia: A Neurocomputational Account of Cognitive Deficits in Medicated and Nonmedicated Parkinsonism. J. Cogn. Neurosci..

[112] Friston K., Schwartenbeck P., FitzGerald T., Moutoussis M., Behrens T., Dolan R.J. (2014). The anatomy of choice: Dopamine and decision-making. Philos. Trans. R. Soc. B Biol. Sci..

[113] Moss J., Bolam J.P. (2008). A Dopaminergic Axon Lattice in the Striatum and Its Relationship with Cortical and Thalamic Terminals. J. Neurosci..

[114] Freeze B.S., Kravitz A.V., Hammack N., Berke J.D., Kreitzer A.C. (2013). Control of Basal Ganglia Output by Direct and Indirect Pathway Projection Neurons. J. Neurosci..

[115] Nambu A. (2004). A new dynamic model of the cortico-basal ganglia loop. Progress in Brain Research.

[116] Wall N.R., Parra M.D.L., Callaway E.M., Kreitzer A.C. (2013). Differential innervation of direct- and indirect-pathway striatal projection neurons. Neuron.

[117] Gertler T.S., Chan C.S., Surmeier D.J. (2008). Dichotomous Anatomical Properties of Adult Striatal Medium Spiny Neurons. J. Neurosci..

[118] Schwartenbeck P., FitzGerald T.H.B., Mathys C., Dolan R., Friston K. (2015). The Dopaminergic Midbrain Encodes the Expected Certainty about Desired Outcomes. Cereb. Cortex.

[119] Schultz W., Dayan P., Montague P.R. (1997). A Neural Substrate of Prediction and Reward. Science.

[120] Haber S.N. (2003). The primate basal ganglia: Parallel and integrative networks. J. Chem. Neuroanat..

[121] Hesp C., Smit R., Allen M., Friston K., Ramstead M. (2019). Deeply felt affect: The emergence of valence in deep active inference. PsyArXiv.

[122] Seamans J.K., Yang C.R. (2004). The principal features and mechanisms of dopamine modulation in the prefrontal cortex. Prog. Neurobiol..

[123] Parr T., Friston K.J. (2019). Attention or salience?. Curr. Opin. Psychol..

[124] Sadeh N., Javdani S., Jackson J.J., Reynolds E.K., Potenza M.N., Gelernter J., Lejuez C.W., Verona E. (2010). Serotonin transporter gene associations with psychopathic traits in youth vary as a function of socioeconomic resources. J. Abnorm. Psychol..

[125] Prosser A., Friston K.J., Bakker N., Parr T. (2018). A Bayesian Account of Psychopathy: A Model of Lacks Remorse and Self-Aggrandizing. Comput. Psychiatry.

[126] Beer J.S., John O.P., Scabini D., Knight R.T. (2006). Orbitofrontal Cortex and Social Behavior: Integrating Self-monitoring and Emotion-Cognition Interactions. J. Cogn. Neurosci..

[127] Somerville L.H., Kelley W.M., Heatherton T.F. (2010). Self-esteem modulates medial prefrontal cortical responses to evaluative social feedback. Cerebral Cortex.

[128] Goossens L., Kukolja J., Onur O.A., Fink G.R., Maier W., Griez E., Schruers K., Hurlemann R. (2009). Selective processing of social stimuli in the superficial amygdala. Hum. Brain Mapp..

[129] Craig M.C., Catani M., Deeley D., Latham R., Daly E., Kanaan R., Picchioni M., McGuire P.K., Fahy T., Murphy D.G.M. (2009). Altered connections on the road to psychopathy. Mol. Psychiatry.

[130] Chavez R.S., Heatherton T.F. (2014). Multimodal frontostriatal connectivity underlies individual differences in self-esteem. Soc. Cogn. Affect. Neurosci..

[131] Chester D.S., Lynam D.R., Powell D.K., DeWall C.N. (2015). Narcissism is associated with weakened frontostriatal connectivity: A DTI study. Soc. Cogn. Affect. Neurosci..

